# Eutectic modification by ternary compound cluster formation in Al-Si alloys

**DOI:** 10.1038/s41598-019-41919-2

**Published:** 2019-04-02

**Authors:** J. Barrirero, C. Pauly, M. Engstler, J. Ghanbaja, N. Ghafoor, J. Li, P. Schumacher, M. Odén, F. Mücklich

**Affiliations:** 1 Functional Materials, Department of Materials Science, Saarland University, D-66123 Saarbrücken, Germany; 2 Nanostructured Materials, Department of Physics, Chemistry and Biology (IFM), Linköping University, SE-58183 Linköping, Sweden; 3 Institut Jean Lamour, UMR CNRS 7198, Université de Lorraine, F-54042 Nancy, France; 4 Institute of Casting Research, Montanuniversität Leoben, A-8700 Leoben, Austria

## Abstract

Al-alloys with Si as the main alloying element constitute the vast majority of Al castings used commercially today. The eutectic Si microstructure in these alloys can be modified from plate-like to coral-like by the addition of a small amount of a third element to improve ductility and toughness. In this investigation the effects of Eu and Yb are studied and their influence on the microstructure is compared to further understand this modification. The two elements impact the alloy differently, where Eu modifies Si into a coral-like structure while Yb does not. Atom probe tomography shows that Eu is present within the Si phase in the form of ternary compound Al_2_Si_2_Eu clusters, while Yb is absent in the Si phase. This indicates that the presence of ternary compound clusters within Si is a necessary condition for the formation of a coral-like structure. A crystallographic orientation relationship between Si and the Al_2_Si_2_Eu phase was found, where the following plane normals are parallel: 011_Si_//0001_Al2Si2Eu_, 111_Si_//6$\bar{7}$10_Al2Si2Eu_ and 011_Si_//6$\bar{7}$10_Al2Si2Eu_. No crystallographic relationship was found between Si and Al_2_Si_2_Yb. The heterogeneous formation of coherent Al_2_Si_2_Eu clusters inside the Si-phase is suggested to trigger the modification of the microstructure.

## Introduction

The use of light-weight castings in the automotive sector results in higher energy efficiency and reduced fuel consumption. Al-Si alloys are frequently used for such castings because of their excellent fluidity, castability, and corrosion resistance^1^. Al-Si alloys have an irregular eutectic phase with faceted Si plates in a non-faceted aluminium matrix. The Si plates act as crack propagation paths that deteriorate the ductility and toughness of the material^2,3^. To improve these properties, the Si plate-like morphology is modified to a coral-like microstructure^4,5^. Small amounts of certain elements such as Na, Sr, or Eu are added to the alloy to completely change the Si-plates to finer, rounded coral branches. The reduced size and geometric aspect ratio of the modified eutectic enhances the toughness of the alloy by reducing the local stress concentrations such that crack initiation is suppressed and crack propagation resistance is increased^3,6,7^. The secondary dendrite arm spacing (SDAS) of the Si-phase is affected by the cooling rate during casting and by combining appropriate casting conditions with additions of modifier and grain refiner, significant improvements in tensile^8–10^ and impact properties^2,6,11^ are obtained.

Understanding the exact underlying mechanism for this remarkable change in structure has been an outstanding research topic ever since the discovery of the modification, almost 100 years ago^12^ and the interaction between Al, Si, and the modifying agents is not yet fully elucidated. Without such knowledge it is impossible to control the homogeneity of the modification for more complex alloys such as Al-Si-Mg and Al-Si-Mg-Cu.

Several studies have considered the effect of the modifier during nucleation and growth of the eutectic phase^13–19^. One of the most distinctive characteristics of the modified Si-phase is the large amount of crystallographic defects^17,20,21^. A well accepted hypothesis by Lu and Hellawell^19^ for the multiplication of defects is the so called impurity induced twinning (IIT). They proposed poisoning of step sources across the closely packed {111} planes in Si by the adsorption of atoms of the modifying agent at the solidification front. They formulated a geometric model to predict the optimal ratio between the atomic radius of the modifying agent and Si such that the adsorption of this impurity on a {111}_Si_ step would cause a displacement resulting in a different stacking sequence and promoting frequent twinning. According to this model the optimal atomic radius of the modifying agent should be 1.65 times larger than the one of Si.

Based on the IIT-model, several elements other than the well-known Na and Sr have been tested as possible alloy modifiers^22–24^. Nogita *et al*.^24^, studied the microstructure of the eutectic phase after addition of almost all rare earth metals and found that, although all of them caused suppressed eutectic growth temperature, the only element able to form the coral-like structure was europium (Eu). In fact, ytterbium (Yb), which is the element with the best fit to the IIT-model (*r*_*Yb*_*/r*_*Si*_ = *1.66*) shows, similar to many other rare earth metals, only a refinement of the Si plate-like structure. This shows that the atomic radius alone is not capable of predicting the morphological transition of the eutectic structure and the reason why they behave differently remains an open question.

Recent studies using atom probe tomography (APT) with the possibility of obtaining spatially resolved chemical information from crystallographic defects in the Si-phase have shown co-segregation of Al atoms and the modifier agent in alloys modified with Sr and Na^25–28^. The defects showed chemical compositions consistent with Al_2_Si_2_Sr and AlSiNa^26^. Based on this, the formation of the coral-like Si structure was proposed to be related to the formation of clusters of ternary compounds at the solidification front rather than the adsorption of single atoms.

The present study investigates and compares the effect of Eu and Yb additions on the microstructure of an Al-Si alloy at different length scales. The aim is to confirm whether the formation of ternary compounds plays a role during the growth of Si corals by Eu addition and highlight the difference of the Si crystal growth with the addition of elements that only refine the Si plate structure such as Yb. Li *et al*.^29^ have shown the presence of Eu at crystallographic defects in the Si phase by high resolution scanning transmission electron microscopy (STEM). The difficulty faced with this technique is that Al atoms in the Si lattice cannot be identified due to the small difference in atomic weight between Al and Si. They also used electron energy loss spectroscopy (EELS) to determine the distribution of Eu atoms within Si but were not able to chemically resolve Al atoms present in the Si crystal. To overcome this problem we used APT, which offers the possibility to study and compare the three-dimensional distribution of Al, Eu and Yb in the Si phase. Transmission electron microscopy (TEM), electron back-scattered diffraction (EBSD), transmission Kikuchi diffraction (TKD) and back-scattered electron (BSE) imaging are also used as complementary techniques to compare the eutectic microstructures at different length scales and to study the orientation relationship between the phases.

## Results

**Figure 1:**
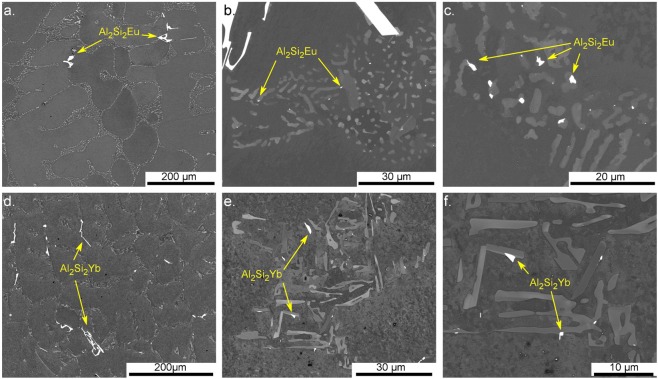
SEM micrographs of Al-5 wt.%Si with either Eu (**a**–**c**) or Yb (**d**–**f**) additions. (**a**) Coral-like eutectic phase and pre-eutectic coarse Al_2_Si_2_Eu intermetallics; (**b**,**c**) smaller Al_2_Si_2_Eu intermetallic phases formed during eutectic growth. (**d**) Refined structure resembling the plate-like eutectic phase of unmodified alloys and coarse Al_2_Si_2_Yb intermetallics; (**e**,**f**) smaller intermetallic phases forming during eutectic growth.

Figure 1 shows two distinct microstructures when adding 0.05 wt.% Eu (Fig. 1(a–c)) or 0.61 wt.% Yb (Fig. 1(d–f)) to an Al-5 wt.% Si alloy. Eu addition results in a homogeneously modified coral-like Si structure, while Yb addition shows less drastic morphological change with a coarser microstructure similar to the plate-like structure of the unmodified alloys. Several studies report that Yb acts just as a refiner of the unmodified plate-like structure^22,24,30,31^. Our results show a mixed structure with straight elongated Si-branches similar to plates together with more rounded ones.

The regions giving bright contrast by backscattered electrons on the polished surfaces are Al_2_Si_2_Eu and Al_2_Si_2_Yb intermetallic phases. The presence of the intermetallic compounds was confirmed by standardless quantitative EDS and their EBSD Kikuchi patterns were successfully indexed to match the crystallographic structure of Al_2_Si_2_Eu and Al_2_Si_2_Yb, respectively^32–34^. Figure 1 shows how these ternary compound particles are present with two different sizes. In Fig. 1(a,d), coarse (tens of micrometres) pre-eutectic intermetallic particles are present, while Fig. 1(b,c,e,f) show particles formed during eutectic solidification with sizes ranging from sub-micrometre to a few micrometres.

**Figure 2:**
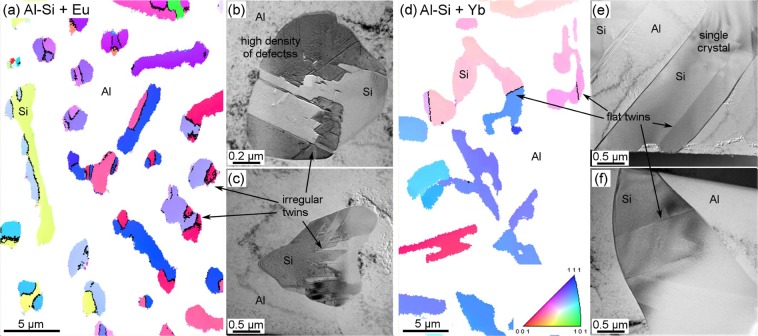
Inverse pole figures of EBSD maps in Al-5 wt.%Si alloys modified by: (**a**) Eu (step size 30 nm) and (**d**) Yb (step size 90 nm). Black lines show twin boundaries in Si. White areas correspond to the Al matrix which was filtered out for clarity. TEM images show high density of crystallographic defects in the Si structure of the Eu modified alloy (**b**,**c**); and only flat twin planes across the Si crystal in the alloy with Yb addition (**e**,**f**).

Figure 2 shows the difference between the eutectic Si structures of the two alloys investigated. Black lines in the EBSD images (Fig. 2(a,d)) highlight irregular Σ3 twin boundaries for Eu addition, which is similar to that in Sr modified alloys^25^. Yb addition shows a lower density of twins in coarser Si branches. The EBSD map in Fig. 2(d) confirms that twin boundaries are straight, which is similar to what have been seen in the unmodified alloy^25^.

The TEM micrographs in Fig. 2 show the crystallographic structure of Si. All micrographs were recorded along the Si<011> zone axis in order to have edge-on alignment of defects on {111} planes. Figure 2(b,c) shows a high density of nanotwins or stacking faults (SF) and v-shaped defects in the alloy with Eu addition, which is consistent with what has been reported for Sr and Na modifications^25,26^. Moreover, the boundaries between the areas with different contrast in the Si phase show irregular shaped twin boundaries. The alloy with Yb addition presents only straight twins through the crystal (Fig. 2(e,f)).

**Figure 3:**
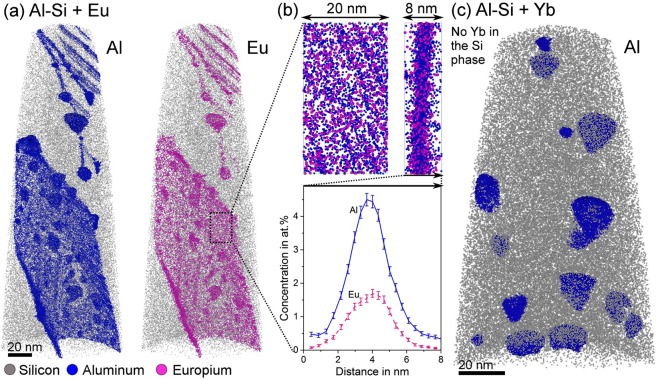
APT comparison of the solute distribution in Si. (**a**) Alloy modified by Eu presenting rod-like, planar and round clusters, all of them containing Al and Eu; (**b**) inset of the planar atomic clustering with a one-dimensional concentration profile across the segregation plane. (**c**) Alloy with Yb addition presenting only Al rounded clusters in Si. No Yb peaks were detected in the mass spectrum of the Si phase.

APT reconstructions in Fig. 3 display differences in the solutes’ distribution in the two alloys. The alloy with Eu addition contains clusters of Eu and Al in the Si structure. Similar to Sr and Na modified alloys^25,26^, solute clusters with three different morphologies are distinguished: rod-like, planar, and rounded (Fig. 3(a)). No clusters containing only Eu or only Al could be found in the Si phase and instead Al and Eu atoms always appear together. Figure 3(b) shows an inset of one such planar cluster of solute atoms with a quantitative one-dimensional concentration profile through the cluster showing the spatial distribution of the atoms. In contrast, no rod-like or planar clusters were found in the alloy with Yb addition (Fig. 3(c)). In fact, no traces of Yb could be detected in the Si phase, i.e. Yb was not even found as solid solution in the Si-crystals. The rounded clusters found in this alloy contain Al and Si, and most of them have a core-shell structure, similar to the unmodified alloy^25^.

**Figure 4:**
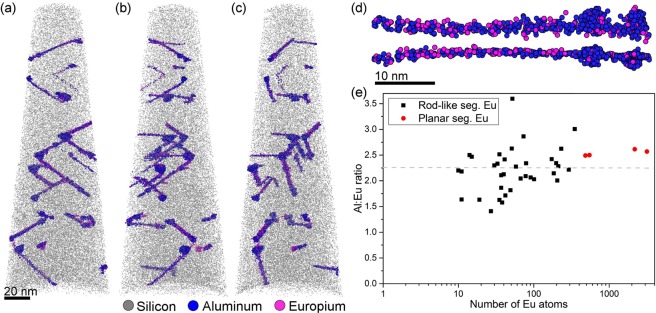
APT analysis of eutectic Si. (**a**), (**b**), and (**c**) are views of the same specimen at different rotation angles showing the scaffold of rod-like segregations in the Si lattice. (**d**) Shows one isolated rod-like segregation viewed from the top and the side. (**e**) Al:Eu ratios as a function of the number of Eu-atoms in each of the 39 clusters (35 rod-like and 4 planar).

To determine the composition of the ternary compound clusters formed in the Eu modified alloy, each single rod-like and planar cluster present in three different APT specimens were isolated in tightly fitted regions of interest (ROIs) and the number of Al and Eu atoms was counted after optimization of the mass spectrum and background subtraction, following the procedure outlined in^26^. The details of a rod-like cluster are shown in Fig. 4(d), and the measured Al:Eu ratios for 35 rod-like and 4 planar clusters are plotted in Fig. 4(e). The average Al:Eu ratio for all these clusters is 2.25 ± 0.42. When considering 2–5 nanometre thick clusters, the atomic positions in APT can be influenced by ion trajectory overlaps due to local magnification effects^35^. This artefact may lead to a convolution of the matrix, in this case Si, with the cluster resulting in an overestimation of the matrix element^35–39^. Given this uncertainty, it is not possible to discriminate between the “matrix Si” and Si in the cluster. Because of this reason, only Al and Eu atoms are considered and reporting relative solute ratios Al:Eu is more adequate than absolute concentrations in the clusters.

Figure 4(a,b,c) shows three perspectives of one APT specimen with a high density of rod-like clusters. All rounded clusters and dissolved solutes in the specimen were masked out to reveal the structure of the rod-like defects. In Fig. 4(a), the specimen is oriented to display the v-shape character of the defects in a similar way to how they are seen by TEM. Figure 4(b,c) are obtained by rotating the specimen. These images reveal the three-dimensional structure of the crystallographic defects in the Si lattice and show how misleading the two-dimensional projection obtained by TEM can be. In several occasions, what we see and denote as v-shaped defects are actually three rod-like clusters originating from one point or two different clusters positioned at different planes in space. Instead of v-shaped defects, APT shows a complex three-dimensional structure of the crystallographic defects in Si. For clarity, videos are included as supplementary material to get complete 3D-views (Supplementary Video S1 and S2).

APT specimens of eutectic Al were analysed and no traces of Eu and Yb were detected with a detection limit of 50 at. ppm showing a negligible solubility of these elements in Al.

The crystallographic orientations between Si and the micrometre sized eutectic Al_2_Si_2_Eu or Al_2_Si_2_Yb phases were analysed by EBSD and TKD. For this analysis, 17 different Si/Al_2_Si_2_Eu boundaries and 10 Si/Al_2_Si_2_Yb boundaries were scanned. Pole figures of the low index plane-normals 001, 011 and 111 in Si were constructed and compared with the pole figures of the low index plane-normals 0001, 11$\bar{2}$0, 01$\bar{2}$2 and 11$\bar{2}$1 in Al_2_Si_2_X (X = Eu,Yb). This comparison allows looking for crystallographic orientation relationships between the ternary phases and the Si crystal. The spatial coincidences of poles from the two phases show parallel plane-normals, or what is the same, parallel planes of the two phases. Alloys modified by Eu showed coincidence of 011_Si_ and 0001_Al2Si2Eu_ poles in 15 out of the 17 cases analysed, while the alloy with Yb addition only showed this orientation relationship in 2 out of 10 cases.

**Figure 5:**
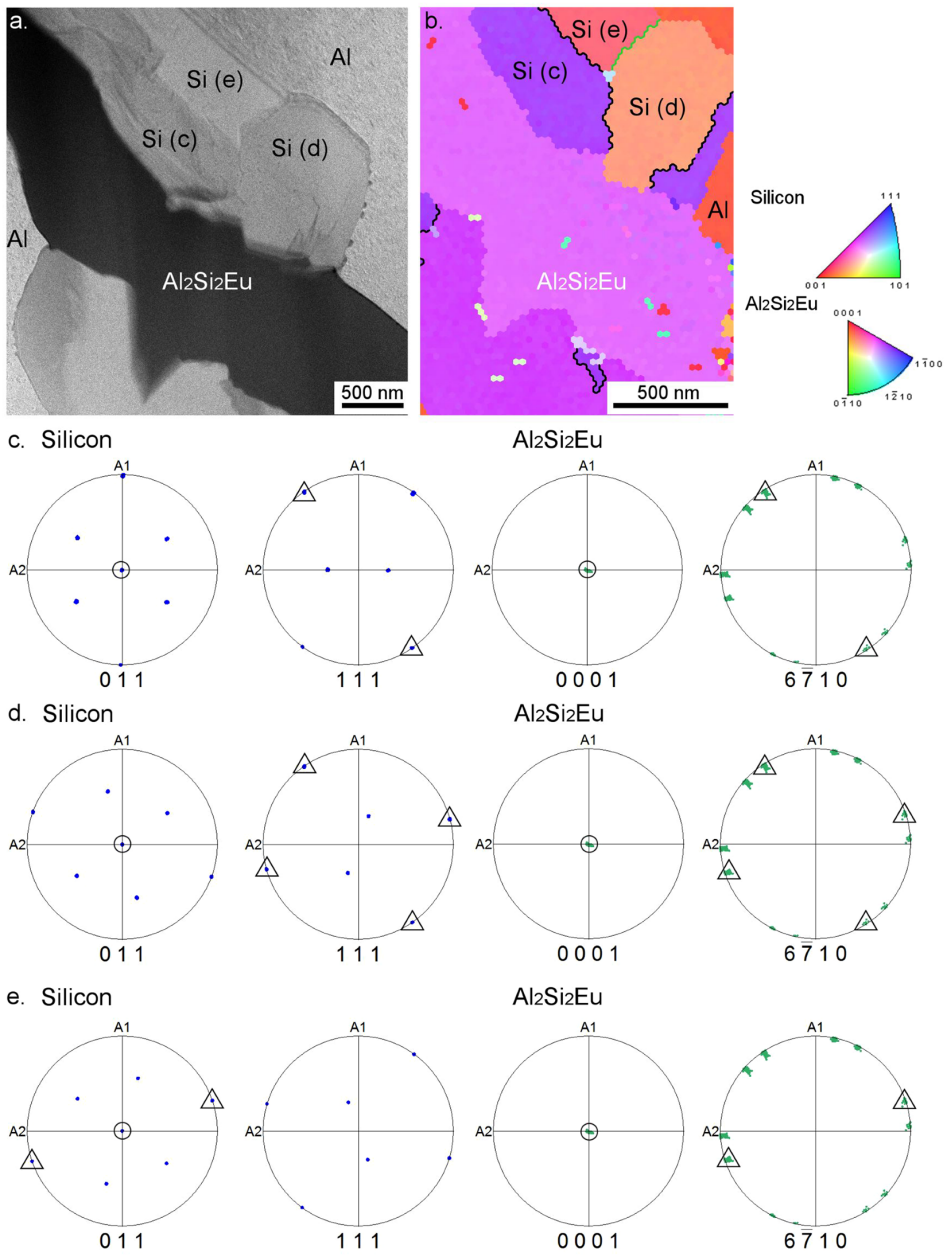
Crystallographic orientation relationship between Si and eutectic Al_2_Si_2_Eu: (**a**) bright field TEM image showing eutectic Al_2_Si_2_Eu surrounded by Si; (**b**) inverse pole figure of a TKD map showing mutually twinned Si crystal orientations. Black boundaries between Si grains show ∑3 twin (60° @<111>), and the green boundary highlights a ∑9 twin (38.9° @<110>). (**c**–**e**) Pole figures (PF) corresponding to the three Si crystals marked in (**b**). All PF were re-oriented to the (0001)_Al2Si2Eu_ in the normal direction (ND) for convenience. Circles depict coincident poles (parallel plane-normals) between 011_Si_ and 0001_Al2Si2Eu_. Triangles depict coincident poles in 011_Si_, 111_Si_ and 6$\bar{7}$10_Al2Si2Eu_.

TKD/EBSD datasets were then rotated to have the 0001_Al2Si2Eu_ plane-normal at the centre of the pole figure, i.e. at the normal direction (ND), to find a further orientation relationship perpendicular to the (0001) _Al2Si2Eu_ lattice plane. Two mutually perpendicular orientation relationships between the crystals define a fixed three-dimensional orientation relationship. Figure 5 shows an example of three Si crystals next to an Al_2_Si_2_Eu phase which fulfil the 011_Si_//0001_Al2Si2Eu_ orientation relationship (Fig. 5(b,c,d,e)). In this condition, pole coincidences depicting parallel plane-normals between the low index {011}, {111} Si plane-families and the {6$\bar{7}$10} Al_2_Si_2_Eu plane-family were determined (Fig. 5(c,d,e)). The simultaneous orientation relationships 011_Si_//0001_Al2Si2Eu_ and 111_Si_//6$\bar{7}$10_Al2Si2Eu_ or 011_Si_//6$\bar{7}$10_Al2Si2Eu_ describe a fixed growth orientation between Si and Al_2_Si_2_Eu which is not present in the case of Si and Al_2_Si_2_Yb.

The three Si crystals shown in Fig. 5 are mutually related by coherent twin boundaries ∑3 and the special grain boundary ∑9 (marked in black and green in Fig. 5(b), respectively). We note that all three Si grain-orientations have parallel low index planes to some plane in the {6$\bar{7}$10} family of the same Al_2_Si_2_Eu grain. (6$\bar{7}$10)_Al2Si2Eu_ plane deviates 7.6° from the low index plane (1$\bar{1}$00)_Al2Si2Eu_.

The misfits of the interplanar spacings between Si and Al_2_Si_2_Eu were calculated to prove the feasibility of a coherent growth of the phases in the orientations found in the pole figures. Table 1 shows misfits smaller than 6% between multiples of the d-spacings. Several other pole coincidences were found involving the Si low index planes and Al_2_Si_2_Eu {12$\bar{3}$0}, {1$\bar{1}$00} and {1$\bar{1}$03} plane-families. Not all coincidences are shown in Fig. 5 for clarity. The relationship between the two mutually perpendicular sets of planes in both lattices is enough to describe the fixed orientation relationship between the crystals.

**Table 1:** Misfits of Al_2_Si_2_Eu related to Si for the parallel planes found in EBSD/TKD.

d-Spacings and multiples (nm)		Misfit δ (%)
	d-{6$\bar{7}$10}Al_2_Si_2_Eu = 0.0552	
**d-{111}Si = 0.314**	(6×) d-{6$\bar{7}$10} = 0.331	−5.19
**d-{011}Si = 0.384**	(7×) d-{6$\bar{7}$10} = 0.386	0.62
	**d-{011}Si = 0.384**	
**d-{0001}Al**_**2**_ **Si**_**2**_ **Eu = 0.726**	(2×) d-{011}Si = 0.768	−5.79

## Discussion

The addition of eutectic modifiers in Al-Si castings affects solidification in several aspects. Eutectic nucleation sites are poisoned causing a depression of the nucleation and growth temperatures, and a strong decrease in the density of eutectic grains^15,40–46^. Fewer and larger grains have a smaller total solid/liquid surface area. To account for a given cooling rate the modified eutectic phase solidifies with a higher interface velocity and, therefore, the microstructure inside the eutectic grains is refined^13,14^. However, the plate-to-coral morphology transition of Si was shown to be independent of alterations in nucleation mode and frequency^47^. The morphology transformation is rather a consequence of an additional effect of the modifier during Si growth. A significant increase of crystallographic defects in Si has been explained by the poisoning of re-entrant twins’ edges^48–52^ and by the impurity induced twinning (IIT) model^19^. The latter points out several atomic species as potential eutectic modifiers of Al-Si alloys^19^. This theory is based on single atoms modifying the structure by poisoning preferred growth orientations. The poisoning atom should optimally be 1.65 times larger than the Si-atom, which would interrupt the growth such that one monolayer in the regular close packed plane stacking sequence is skipped and instead a twin is formed.

Today we know that, although the modifier agent influences the formation of crystallographic defects, the atomic radius itself cannot be taken as an indication of potency of modification. This is exemplified as just some of the elements proposed in IIT form Si-corals. For example, Yb has the ideal ratio r_Si/_r_Yb_ = 1.65 but it only refines the plate-like Si structure^22,30,31^, while Eu having a ratio of 1.72 is as potent as Na and Sr to form Si-corals^30,53^. Further, in this paper we show that an efficient modifier like Eu always appears together with Al in the defects of the Si lattice (Figs 3 and 4) and never as single atoms. This is in accordance with other efficient modifiers such as Sr and Na, which showed compositions inside the defects consistent with Al_2_Si_2_Sr and AlSiNa^25,26^. In the present work, the average Al:Eu ratio of nearly 2 (Fig. 4(e)) suggests that the ternary compound Al_2_Si_2_Eu is formed at the defects and in this case the geometric IIT model is no longer valid. The presence of clusters of Al_2_Si_2_Eu at the defects in the Si lattice stresses the importance of atomic interaction between Si, the third element (in this case Eu), and Al during solidification.

The formation of the eutectic ternary compounds Al_2_Si_2_Eu and Al_2_Si_2_Yb (Figs 1(b,c,e,f); 5(a)) indicates that there is sufficient driving force for nucleation and growth of these phases during solidification. To the best of our knowledge, the liquidus surfaces for Al-Si-Eu and Al-Si-Yb ternary systems are not known. However, the liquidus surfaces for alloys with other modifiers such as Al-Si-Sr^54^, Al-Si-Na^55^ and Al-Si-Ca^56^ show a ternary eutectic point close to the temperature and composition of the binary Al-Si and at low amounts of Sr (0.03 at.%), Na (0.01 at.%), or Ca (0.7 at.%). Based on the back-scattered-electron images in Fig. 1 and other studies^29–31,53^, it is reasonable to assume that Al-Si-Eu and Al-Si-Yb alloys also present such eutectic point. At the ternary eutectic point, three phases are expected: Al, Si, and a ternary compound inherent to each system.

Examining the ternary eutectic solidification path and keeping in mind that the amount of the third element (Eu, Yb) in the alloy is low, there is a simultaneous solidification of the two major phases (Al and Si) during the entire eutectic reaction. The third phase will only form when the third element’s local concentration is high enough. APT results show that almost no Eu or Yb is dissolved in the eutectic Al or Si. Instead, these elements are expected to be accumulated ahead of the solidification front resulting in a concentration gradient of Eu or Yb perpendicular to the growth front. Such accumulation of the modifying element has been proposed in several studies^50,57–59^ and it gives the necessary conditions of constitutional undercooling and supersaturation for ternary compounds to form. The Gibbs free energy for nucleation of embryos is a function of the undercooling and the local supersaturation of the elements. At the eutectic temperature, the diffusion boundary layer at the solid/liquid interface has an increased amount of the third element causing the melt to locally be more undercooled^22,31,53^. Under these conditions, sub-critical clusters (embryos) of the ternary phase will form and re-melt in the liquid until there is enough supersaturation for a cluster to reach a critical size and grow to a micrometre-sized grain as seen in Fig. 1(b,c,e,f). Figures 1(c,f) and 5(a) highlight how the eutectic ternary phase is formed on the Si phase and do not grow independently or as a part of eutectic Al. This observation emphasizes that these phases are most probably heterogeneously nucleated at the Si interface. This is reasonable because the Al_2_Si_2_X phases normally have an ordered hexagonal structure (space group P-3m1 (164)) and grow in a faceted manner such as Si. This tendency towards faceted growth can be seen in the polyhedral morphology of the pre-eutectic coarse particles (Fig. 1(a,d)).

The microstructural comparison between the two alloys studied here shows that they have similar solidification paths. Both alloys have a ternary eutectic point with formation of Al_2_Si_2_X (Eu,Yb) (Fig. 1). The difference between the two alloys is Eu’s ability to form early stage Al_2_Si_2_Eu clusters on the facets of the Si crystal, while such clusters are absent in the alloy containing Yb (Figs 3 and 4). The formation of clusters on the Si-facets during solidification affects the Si-growth by local obstruction, causing steps on the facets and increasing growth direction diversity. This growth restriction model is different to the IIT model, as it incorporates the much larger ternary clusters as growth obstacle on the Si surfaces explaining more readily the high density of crystallographic defects in Si and the modification from Si plates to corals.

We postulate that the difference between the effect of Eu and Yb stems from the difference in the energy needed for sub-critical clusters of Al_2_Si_2_Eu and Al_2_Si_2_Yb to form on solidified Si surfaces. In other words, Al_2_Si_2_Eu clusters need less energy to form on Si in comparison to Al_2_Si_2_Yb clusters. The strong accumulation of the modifier together with Al and Si on the first tens of nanometres ahead of the solidification front, gives the necessary condition of constitutional undercooling and supersaturation for the ternary clusters to form. Eu and Yb atoms will be eventually trapped at the Si surface as part of ternary clusters formed heterogeneously at the interface. However, the cluster formation is only energetically favourable for a good lattice match onto the Si surface. The presence of Al_2_Si_2_Eu clusters showed by APT suggests a good lattice match with Si, in contrast to Al_2_Si_2_Yb. Although Yb atoms may sit shortly onto the Si interface, the lattice mismatch of the ternary cluster cost too much energy and dissolves or cannot form.

Direct determination of the formation energy of heterogeneous clusters of Al_2_Si_2_Eu and Al_2_Si_2_Yb on different facets of the Si crystal during eutectic solidification is challenging, both experimentally and by simulations. Instead, we have adopted an indirect approach to test our arguments by studying the crystallographic orientation relationships between Si and each of the two ternary phases. The expected crystallographic orientation relationship between fcc and hcp is the alignment of the close packed planes 111_fcc_//0001_hcp_. However, Fig. 5 shows EBSD/TKD pole figures of Al_2_Si_2_Eu in contact with Si, showing unexpected parallel plane-normals: 111_Si_//6$\bar{7}$10_Al2Si2Eu_, 011_Si_//6$\bar{7}$10_Al2Si2Eu_ and 011_Si_//0001_Al2Si2Eu_ with misfits of the d-spacings smaller than 6% (Table 1). This result indicates heterogeneous formation of Al_2_Si_2_Eu with an epitaxial relationship to the Si surface, while such relationship is not present in the case of Al_2_Si_2_Yb. Both ternary compounds, Al_2_Si_2_Eu and Al_2_Si_2_Yb, have the same hexagonal P-3m1 crystal structure with a = b lattice parameters that differ less than 1%, while c differs more than 4% (Table 2). Considering the orientation relationship reported in Fig. 5, the different potency of heterogeneous nucleation for these two phases on Si is evaluated on the basis of the misfit between the Si-surface and the nucleating phases. The interplanar spacings d(0002)_Al2Si2Eu_ = 0.363 nm and d(0002)_Al2Si2Yb_ = 0.348 nm can be compared to d(110)_Si_ = 0.384 nm. This comparison shows misfits of 5.79% for Al_2_Si_2_Eu and 10.50% for Al_2_Si_2_Yb towards Si. The higher mismatch of the Yb phase adds strain energy to its nucleation, which makes Al_2_Si_2_Yb clusters less stable than Al_2_Si_2_Eu clusters on Si facets.

**Table 2:** Crystal structure and misfit of ternary compounds related to Si.

	Space group	Lattice parameters		d-spacing (nm)	Misfit δ %	Si structure
		a,b (nm)	c (nm)			
**Si**	**Fd-3m (227)**	**0.543**	**0.543**	**d(110) = 0.384**	—	—
Al_2_Si_2_Sr^63^	P-3m1 (164)	0.418	0.743	d(0002) = 0.372	−3.36	Coral
AlSiNa^64^	P4/nmmO2 (129)	0.414	0.738	d(002) = 0.369	−4.07	Coral
**Al**_**2**_ **Si**_**2**_ **Eu** ^33^	**P-3m1 (164)**	**0.418**	**0.726**	**d(0002) = 0.363**	**−5.79**	**Coral**
Al_2_Si_2_Ca^63^	P-3m1 (164)	0.413	0.715	d(0002) = 0.358	−7.41	Mixed
Al_2_Si_2_Ba^65^	I4/mmm (139)	0.423	0.698	d(0002) = 0.349	−10.03	Mixed
**Al**_**2**_ **Si**_**2**_ **Yb** ^33^	**P-3m1 (164)**	**0.414**	**0.695**	**d(0002) = 0.348**	**−10.50**	**Refined plates**
Al_2_Si_2_Y^66^	P-3m1 (164)	0.418	0.656	d(0002) = 0.328	−17.07	Refined plates

This calculation can be applied to similar ternary phases present in the Al-Si alloys with addition of other elements. Table 2 shows how the three systems forming Si corals (addition of Na, Sr or Eu), have ternary phases with a misfit smaller than 6%. Ca and Ba, elements which can induce the formation of fibrous Si similar to the coral structure when higher amounts of these elements are added^22,23^, have a higher misfit, while Yb and Y ternary phases show the highest misfits and also the lowest potency as modifiers^22,31^.

The reason why clusters with a rod-like morphology are the most frequently found is likely an effect of different misfits in different orientations on the Si-surface. Such dispersion in misfit favors growth of the cluster in specific orientations. These results show a consistent difference between the elements tested as possible modifiers in literature and the degree of Si microstructural modification into corals. Li *et al*.^29^ showed by high resolution STEM imaging how columns of Eu atoms match every second Si pair of dumbbells on a 111_Si_ plane. The approximated distance between Eu columns that can be measured from their image is ~0.7 nm, i.e. the c distance of Al_2_Si_2_Eu. Al and Si atoms between Eu columns cannot be differentiated in HAADF-STEM mode because of the projection of the entire thickness of the TEM sample on the image (about 20 nm with more than 20 Al and Si atomic layers) and because of the small Z difference between Al and Si. However, the distance between Eu columns can be explained by the early stage formation of atomic layers of the Al_2_Si_2_Eu ternary compound on a 111_Si_ plane as postulated in this work.

## Conclusion

- This study shows clusters of Eu co-segregated with Al in eutectic Si matching the stoichiometry of Al_2_Si_2_Eu ternary compound. On the contrary, no Yb in the Si crystal was found. This result suggests that the existence of nanometre sized clusters of ternary compounds is a necessary condition for the formation of a coral-like structure.
- The formation of Al_2_Si_2_Eu and Al_2_Si_2_Yb during eutectic solidification proves the presence of a ternary eutectic reaction for these alloys.
- The parallel lattice plane-normals 011_Si_//0001_Al2Si2X_, 011_Si_//6$\bar{7}$10_Al2Si2X_ and 111_Si_//6$\bar{7}$10_Al2Si2X_ found only for the sample with Eu addition and not for Yb proves a favourable heterogeneous formation of Al_2_Si_2_Eu on Si. This explains the formation and adsorption of clusters of this phase in the alloy with Eu addition and not in the alloy with Yb addition.
- The misfit between 011_Si_ and 0002_Al2Si2X_ interplanar spacings shows a consistent trend with the potency of modification for several elements such as Sr, Na, Eu, Ca, Ba, Yb and Y.

In the present work, the growth restriction of eutectic Si by the formation of ternary clusters is proposed. The formation of such clusters on Si-facets creates growth steps increasing growth direction diversity. The incorporation of cluster onto the Si surface explains the high density of crystallographic defects in Si and the modification from Si plates to corals.

## Methods

Ingots of about 1 kg Al-5Si-0.05Eu and Al-5Si-0.61Yb alloys (wt. %) were produced by electric resistance melting of the charge material in a boron-nitride coated clay-graphite crucible at 750 °C and then casted using gravity die casting. Prior to casting, no degassing treatment was performed. High purity Al (5N) and Si (5N) were used as starting materials. Master alloys, Al-2Eu and Al-5Yb, were used to add Eu and Yb when the starting materials (Al and Si) were fully molten. The modifier amounts were chosen to yield optimal modification based on previous studies^22,24,29,31,53^.

Microscopy samples were prepared from the casts by metal machining, followed by grinding and polishing to a mirror-like finish, where the last step was vibrational polishing in a colloidal silica suspension (Struers OP-S - 0.04 µm) for 2 hours. TEM and APT samples were prepared in a dual-beam focused ion beam/scanning electron microscopy workstation (FIB/SEM) (Helios NanoLab 600™, FEI Company, USA). APT specimen of the eutectic Si phase were prepared by the phase selective sample preparation method described in^60^. After lift out and thinning of the samples, a low energy milling at 2 kV was performed to minimize gallium induced damage^61^.

An EDAX Hikari detector within the FIB/SEM workstation was used to record EBSD and TKD data using an electron beam of 20 kV/22 nA and 30 kV/5.5 nA, respectively. Al_2_Si_2_Eu and Al_2_Si_2_Yb crystal structures from literature^32–34^ were added to OIM Data Collection 7 database by means of the built-in structure creation wizard. Post-processing of the data was performed using the OIM Analysis software (EDAX). Results are shown in the form of pole figures and inverse pole figure maps. In case of the TKD measurement shown in Fig. 5, a pseudosymmetry clean-up followed by a grain dilation was performed for visual representation. Pole figures were calculated from uncleaned data. Conventional EBSD scans in reflection mode yielded better pattern quality. Data points for pole figure calculation were extracted from uncleaned raw data using the grain highlight function of OIM Data Analysis software. This allows a point-and-click selection of data points belonging to a continuous grain based on a misorientation threshold (here 5°). Data points from the intermetallic phase (Al_2_Si_2_Eu or Al_2_Si_2_Yb) and the neighbouring Si phase were highlighted and used for the calculation of pole figures. No clean-up was used.

TEM and STEM imaging was performed in a Tecnai G2 TF 20 UT FEG (FEI) in micro and nanoprobe modes and a JEM - ARM 200 F Cold FEG TEM/STEM operating at 200 kV and equipped with a spherical aberration (Cs) probe and image correctors (point resolution 0.12 nm in TEM mode and 0.078 nm in STEM mode).

APT was carried out in a LEAP 3000X HR (CAMECA) in laser mode to measure eutectic Si and voltage mode to measure eutectic Al. All measurements were performed at repetition rate of 200 kHz, pressure lower than 1.33 × 10^−8^ Pa, and evaporation rate of 5 atoms per 1000 pulses. Laser-pulsed APT was accomplished using a laser with a wavelength of 532 nm, pulse length of 10 ps, and a pulse energy of 0.3–0.4 nJ while keeping a specimen temperature of about 40 K. Voltage measurements were performed at 20% pulse fraction and a specimen temperature between 50 and 60 K. Datasets were reconstructed and analysed with IVAS™3.6.8 software (CAMECA). Al and Eu contents in Si were measured after background subtraction performed with IVAS software. The cleaning of the datasets for visualization purposes was performed with the open source software Blender 2.76 and the open access AtomBlend plugin developed by Peter Felfer and Vavara Efremova^62^.

## Supplementary information

Supplementary information

Video S1

Video S2

## Data Availability

The datasets analysed during the current study are available from the corresponding author on reasonable request.
