# Supplementary material for: Eutectic modification by ternary compound cluster formation in Al-Si alloys
Source: Sci Rep. 2019 Apr 2;9:5506. doi: 10.1038/s41598-019-41919-2 (PMC6445082; doi:10.1038/s41598-019-41919-2)
Supplement: Supplementary file 1 — Supplementary information [file 41598_2019_41919_MOESM1_ESM.docx]

Eutectic modification by ternary compound cluster formation in Al-Si alloys

J. Barrirero^a,b^ *, C. Pauly^a^, M. Engstler^a^, J. Ghanbaja^c^, N. Ghafoor^b^, J. Li^d^, P. Schumacher^d^, M. Odén^b^, F. Mücklich^a^

^a^ Functional Materials, Department of Materials Science, Saarland University, D-66123 Saarbrücken, Germany

^b^ Nanostructured Materials, Department of Physics, Chemistry and Biology (IFM), Linköping University, SE-581 83 Linköping, Sweden

^c^ Institut Jean Lamour, UMR CNRS 7198, Université de Lorraine, F-54042, Nancy, France

^d^ Institute of Casting Research, Montanuniversität Leoben, A-8700, Leoben, Austria

Supplementary information

For clarity, videos showing 3D views of the APT reconstructions in Figures 3 and 4 in the paper are included as supplementary material (Supplementary Video S1 and S2).

**Video legends**

Supplementary Video S1: APT 3D-view of the eutectic Si phase in Eu modified alloy. Si = gray; Eu = pink; Al = blue.

Supplementary Video S2: APT 3D-view of the eutectic Si phase in Eu modified alloy. Si = gray; Eu = pink; Al = blue.
